# Over Time Decay of Cortisol Metabolites in Faecal Pellets of Koalas in Central Queensland

**DOI:** 10.3390/ani11123376

**Published:** 2021-11-25

**Authors:** Flavia Santamaria, Rolf Schlagloth, Rupert Palme, Joerg Henning

**Affiliations:** 1 Koala Research-Central Queensland and Flora, Fauna and Freshwater Research, School of Health, Medical and Applied Sciences, Central Queensland University, North Rockhampton, QLD 4702, Australia; r.schlagloth@cqu.edu.au; 2 Department of Biomedical Sciences, University of Veterinary Medicine, 1210 Vienna, Austria; Rupert.Palme@vetmeduni.ac.at; 3 School of Veterinary Science, The University of Queensland, Gatton, QLD 4343, Australia; j.henning@uq.edu.au

**Keywords:** koala, *Phascolarctos cinereus*, glucocorticoid metabolites, faeces, stability, non-invasive monitoring, stress, decay

## Abstract

**Simple Summary:**

Faecal cortisol metabolites (FCMs) are a useful, non-invasive tool for the assessment of stress in koalas. However, FCM stability after defecation is a critical issue. Therefore, we exposed faecal pellets of koalas to three different environmental conditions and measured FCMs with three recently evaluated enzyme immunoassays (EIAs). Because water loss over time exerted the greatest influence on FCMs, we strongly recommend collecting freshly defecated pellets in koalas.

**Abstract:**

Faecal material can be a valuable source of information for a range of animal health aspects and can be used to measure faecal cortisol metabolites (FCMs). FCM values can relate to physiological stress responses. However, freshly defecated pellets are not always available and environmental conditions, such as temperature and humidity, might affect faecal pellet consistency and FCM levels. Therefore, the impact of environmental conditions on FCMs needs to be evaluated. We collected 107 pellets from two female and two male koalas, exposed them to three types of treatments, and analysed FCMs in these samples with three enzyme immunoassays (EIAs). After analysis, the original FCM values were mathematically corrected for water loss. Results show that the FCMs were more stable when measured using tetrahydrocorticosterone (50c) and 5α-pregnane-3β,11β,21-triol-20-one (37e) EIAs, and were less stable when measured with the cortisol EIA. With 50c, the FCM values did not vary significantly over time either before or after the adjustment with water in the environment treatment group. For samples kept under constant low (25 °C) and high (35 °C) temperatures, the 50c FCM values did not vary significantly over time, after adjustments were made for water loss. Thus, this study highlights the importance of considering the suitability of faecal field samples for FCM analysis. Because water loss was the main driver of FCM changes, we strongly recommend collecting koala pellets that are freshly defecated, despite the effort and time it might take to collect such pellets.

## 1. Introduction

Capturing and handling wildlife without causing stress is a difficult task. Therefore, scientifically valid and ethically acceptable methods that do not impact the health of animals need to be explored. There has been an increase in the development of non-invasive methods using faecal material to obtain information on the wellbeing of wild or captive wildlife [1,2,3,4]. Collecting faecal pellets from the ground avoids stress associated with catching animals and obtaining blood samples.

Faecal material can be used to measure faecal cortisol metabolite (FCM) values [5,6], which are an indicator of adrenal activity and can be related to physiological responses to stressors [2]. Currently, the use of fresh faecal samples is recommended for this type of analysis [5,6,7,8,9]. However, freshly defecated pellets are not always available in field studies. Environmental conditions including, but not limited to, temperature and humidity affect faecal bacterial activity and cortisol metabolites [10,11,12].Excreted FCMs and microbiomes are also species-specific, and the method used for measuring the metabolites plays a crucial role. Therefore, the stability of FCMs needs to be evaluated for each species and method [2,11].

The survival of the koala, an iconic Australian marsupial, is currently threatened by a number of stressors that are often related to human activities, such as habitat fragmentation [13,14]. In Queensland, Australia, the koalas’ distribution stretches from the south-east to the northern part of the state [15]. We are currently conducting research, including FCM studies, that explore non-invasive investigations of the health status of koalas in Central Queensland (CQ). Central Queensland extends around the Tropic of Capricorn, from the east coast of Queensland to the Northern Territory in the west, and it includes several bioregions. The two most important bioregions where fragmented koala habitat is found are the CQ coast bioregion and part of the Brigalow bioregion. The climate of this area is sub-tropical; summers are characteristically humid and hot, and winters are typically warm and dry [16].

Thus, the decay of cortisol metabolites in faecal samples under varying temperature and humidity needs to be considered and is investigated in the study presented here. Moreover, we weigh up the benefit of collecting freshly defecated faeces (pellets) despite the effort and time it takes to collect such samples.

## 2. Materials and Methods

This study was conducted between 29 May and 13 June 2021 (Australian winter).

### 2.1. Animals and Sample Collection

At least 10 pellets from defecations of two male (Rockhampton Zoo, Rockhampton, Queensland) and two female koalas (Cooberrie Park, Woodbury, QLD, Australia) were collected across a number of days and used in three treatments (E, H or L; see below). On one occasion, only 5 pellets could be collected from one of the females. A hollow mat was positioned under the tree stumps to prevent the pellets falling on sand and possible contamination with urine. Pellets were placed in plastic cups and labelled with ID codes, which included type of treatment, sex (F or M), koala number (1 or 2), a number if treatment was repeated (1 or 2), as well as the date. These ID codes and the times of collection were recorded on a spreadsheet.

At day 0, each pellet was weighed and two pellets from each defecation, labelled with all the details listed above, were placed into a test tube and stored in a freezer (−20 °C). The remaining pellets of each defecation were placed in the allocated treatments in the same order on the ground (E) or on the trays (L and H) on which they were weighed.

Thereafter, for each treatment, a sample consisting of two pellets per day (or one if fewer pellets were available in the defecation) from each defecation were collected at day 1 (24 h), 2 (48 h), 4 (96 h), and 7 (168 h). At each time point, two pellets (or one) were removed from each treatment and their weight was recorded. The two pellets were then placed into a test tube and stored in the freezer (−20 °C). In total, 107 samples were analysed for FCMs in this study.

### 2.2. Treatments

The pellets were exposed to three treatments, E, L, and H:

- E (environment): The pellets were laid on the ground on leaf litter under trees. A basket with five walls made of chicken wire mesh was placed over the pellets to protect them from mechanical disturbance, while allowing exposure of the pellets to local weather conditions. A digital datalogger (USB Temperature/Humidity Datalogger with LCD QP6014, Jaycar Electronics, Sydney, Australia), placed adjacent to the samples and covered by a makeshift roof, recorded temperature (T) and relative humidity (RH) at 10 min intervals over the 7-day treatment period. The pellets were exposed to any weather event occurring during the study. Plant tags with ID codes marked each sample. At the day of sample collection, photos of the whole defecation and of the pellets removed were taken to provide a visual record of the experiment.
- L (standard ambient T-25 °C): The pellets were placed in an incubator set at a constant temperature of 25 °C. RH was monitored with a wet/dry thermometer.
- H (high T and high RH): The pellets were placed in an incubator set at a constant temperature of 35° C. A tray of water was placed inside to generate high humidity. A digital datalogger (Digitech XC-0424, Jaycar Electronics, Sydney, Australia) positioned inside the incubator recorded T and RH at 10 min intervals over the 7-day treatment period.

T and RH in treatments L and H represented conditions similar to the subtropical winter and summer, respectively, without the variations of night and day, wind, shade, sun, and rain that samples were exposed to under treatment E.

### 2.3. FCM Analysis

The extraction procedure and analyses are described in Santamaria et al. [6]. In brief, 500 mg of ground samples, neither treated by heat nor freeze-dried, was placed into a 10 mL tube and 5 mL of 80% methanol was added. Samples were shaken for 30 min with an orbital rotator shaker, vortexed for 2 min with a hand vortex, and centrifuged at 2500 g for 15 min. Completely dried down aliquots (0.25 mL) of the extracts in 1 mL Eppendorf tubes, sealed with paraffin film, were shipped to the University of Veterinary Medicine (Austria), where dried sample extracts were resolubilised in 80% methanol and a further dilution step with assay buffer (1 + 9). Aliquots were analysed in duplicate with the cortisol (37e) and tetrahydrocorticosterone (50c) enzyme immunoassays (EIAs), as described in Santamaria et al. [6,9].

### 2.4. Water Loss

Based on the change in weight between day 0 and the day when pellets were collected for storage in the freezer, the percentage of water loss for each pellet and the average percentage of water loss for the two pellets used for the analysis were calculated. After samples were analysed with the three EIAs, the percentage water loss for each analysed sample was mathematically added to the ‘original’ FCM value (ng/g) to obtain the ‘adjusted’ FCM value using the following equation:

Adjusted EIA value = original EIA value −[(original EIA value × % water loss) / 100]

Calculations were conducted in Microsoft Excel.

### 2.5. Statistical Analysis

The impact of time (‘days since commencement of the treatment’) on ‘water loss’ in fecal pellets was evaluated in separate generalised estimating equation (GEE) models for each treatment. Analysis was conducted with ‘koala ID’ considered as grouping variable. A Gaussian distribution with an identity link and an exchangeable correlation structure was specified for the GEE models. A Wald test was used to test the overall significance of the categorical variable ‘days since commencement of the treatment’.

The Spearman correlation coefficient was used to describe the correlation between ‘mean water loss’ and the ‘range of T and RH values between two successive samplings’.

Mixed effect linear regression, with ‘koala ID’ as grouping variable or random effect and time (‘days since commencement of the treatment’) as fixed effect, was used to evaluate the change of ‘original’ and ‘adjusted FCM’ values over time. A restricted maximum likelihood (REML) approach was used for the fitting of the linear mixed models. Again, a Wald test was used to test the overall significance of the categorical variable ‘days since commencement of the treatment’. Data analysis was conducted in Stata 17.

## 3. Results

### 3.1. Temperature and Relative Humidity

**Figure 1:**
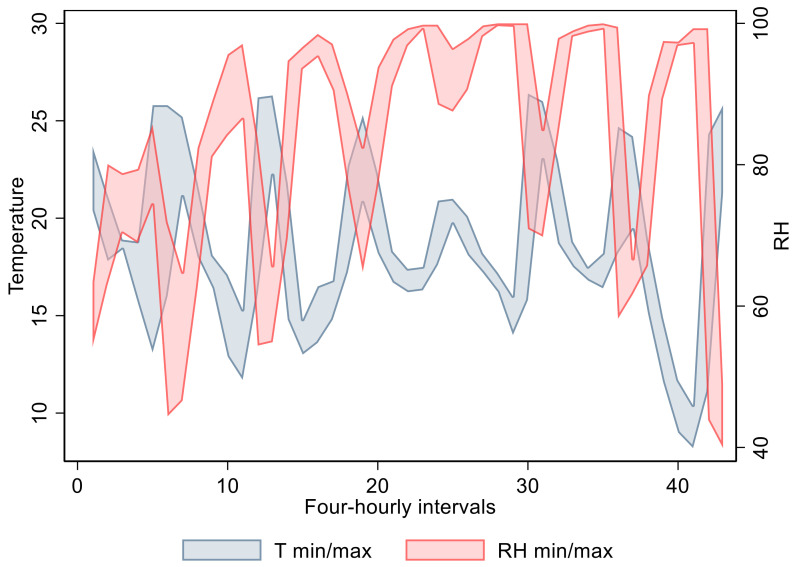
Range of T (temperature in °C) and RH (relative humidity in %) during the seven-day period of treatment E.

E treatment: The median T over the duration of the trial was 18 °C (range: 8.2–26.4 °C), whereas the median RH was 90.2% (range: 39–100%). Figure 1 displays the range of T and RH values over the seven-day treatment period divided into four-hourly intervals. RH reached values close to 100% during a substantial rain event during the night between day 3 and 4 and during day 4.

L treatment: T was held constant at 25 °C and RH was constant at 50%.

H treatment: T was held constant at 35 °C; the median RH was 90% (range: 60–96%)

### 3.2. Water Loss

**Figure 2:**
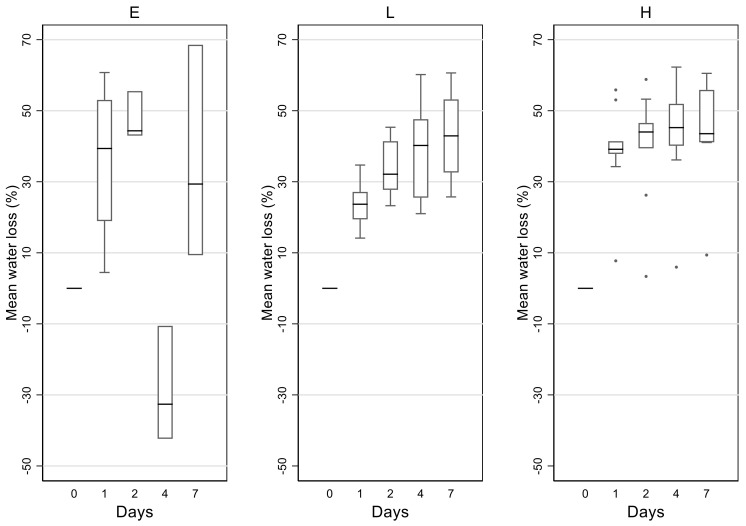
Box and whisker plots for percentage water loss (relative to day 0) over time for koala faecal samples under the three treatments E (environment), L (standard ambient T, 25 °C), and H (high temperature, T and high relative humidity, RH).

The mean water loss for samples for all three treatments is shown in Figure 2. For treatment E, after the rain event during the night of day 3 to day 4 and on day 4, samples collected on day 4 were heavier than at day 0, indicating substantial absorption of water. Water loss between day 0 and day 1 was considerably higher under treatment H compared to treatment L, as samples under treatment H were exposed to a higher temperature.

Modelling the mean water loss in three separate GEE models for each treatment indicated significant changes in water content over time for each treatment (*p* < 0.001). Although water loss fluctuated for E (mainly due to the rain event specified, resulting in negative water loss on day 4), water loss increased over time for L and H treatments.

The relationship between climate variables and water loss (or gain) in pellets for all three treatments was explored. Supplementary Figure S1 shows, for treatment E, the relationship between water loss in two successive sample periods and the range of T and RH values for these successive samplings. There was a moderate correlation between mean water loss and ranges of T (Spearman Rank = 0.62, *p* = 0.025) and RH (Spearman Rank = 0.55, *p* = 0.053) values between two successive samplings for samples under treatment E.

There was also minimal variation in RH values for H, and no significant correlation was detected between mean water loss and RH (Supplementary Figure S2). As the temperature in the incubator for L and H, and the relative humidity for L remained constant, correlation coefficients could not be calculated. 

### 3.3. Original and Adjusted FCM Values

**Figure 3:**
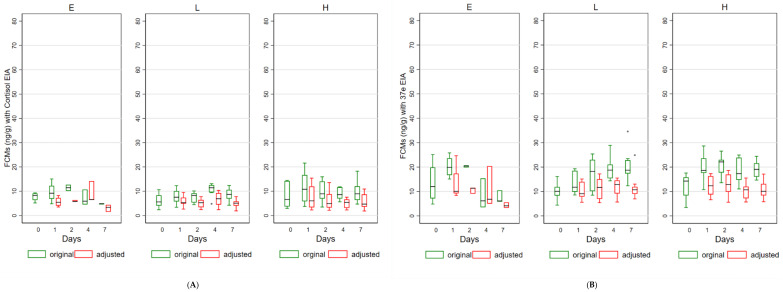

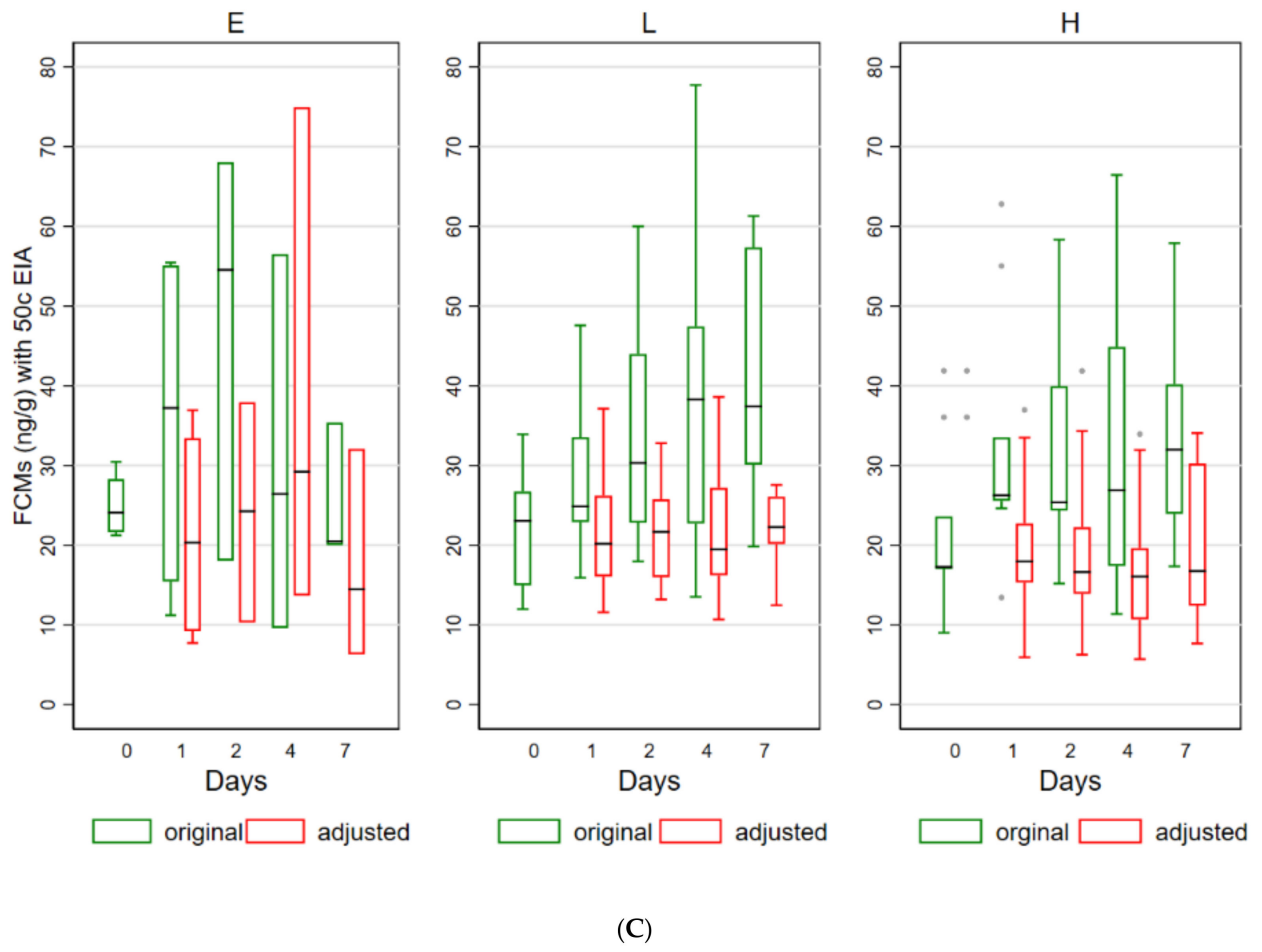
Box and whisker plots for original and adjusted FCM (faecal cortisol metabolite) values measured by the cortisol (**A**), the 37e (**B**), and the 50c (**C**) EIAs (enzyme immunoassays) over time in the three treatments E (environment), L (standard ambient T, 25 °C), and H (high temperature, T and high relative humidity, RH).

Figure 3, illustrates both original and adjusted FCM values measured by the cortisol, the 37e and the 50c EIAs for the three treatments over time. The range of FCM values detected by the cortisol and 37e EIAs was lower compared to the range detected by the 50c EIA.

Using a mixed effects model analysis with ‘koala ID’ as a random effect, it was shown that original values measured by the cortisol EIA varied significantly over the sampling period of 7 days under treatments E (*p* = 0.0314) and L (*p* = 0.0017), but not H (*p* = 0.3147). Original values measured by the 37e EIA varied significantly over time under all treatments (E: *p* = 0.0003, L: *p* < 0.0001, H: *p* < 0.0001). A significant variation in original values measured by the 50c EIA over time was identified for treatments L (*p* = 0.0001) and H (*p* < 0.0001), but not for E (*p* = 0.3301). After the mathematical correction for change in water content, the adjusted FCM values measured with the cortisol EIA still indicated significant variation over time for treatment E (*p* = 0.0225), but not for L (*p* = 0.2113) and H (*p* = 0.2187). Adjusted FCM values measured with the 37e EIA showed no significant variation over time (E: *p* = 0.3790, L: *p* = 0.7358, H: *p* = 0.3287). No significant variation in adjusted values measured with the 50c EIA over the sampling period was identified for treatment E (*p* = 0.4035), L (*p* = 0.9996), and H (*p* = 0.4389). Overall, FCMs were more stable when measured with the 50c and 37e EIAs, and water loss was the main driver for the observed variations in the original FCM values.

Descriptive statistics for original and adjusted FCM values measured by the 50c EIA are shown in Supplementary Table S1.

## 4. Discussion

The effect of enzymes produced by the intestinal microbiome on the metabolism of steroids is well known, and studies have shown that the quality and quantity of metabolites are influenced by intestinal bacteria [2,12,17]. The effect of bacteria continues in faecal material after defecation and can have effects on the stability of metabolites [18].

Methods used for establishing the decay of faecal glucocorticoid metabolites need to be specific to the species being studied [2]. We used the same method used by Santamaria et al. [6,9] to measure FCMs in koala. Hence, pellets were neither heat treated nor freeze-dried, for consistency. However, we used a novel approach where the percentage of water loss over time was recorded and mathematically considered to adjust the original EIA values. This allowed us to determine whether changes to the FCM values in the adjusted samples were due to decay or the increased concentration in drier faecal samples collected after day 0. In fact, although the weight of the samples used for analysis was consistently 0.5 g, drier samples throughout the treatment had less water (more faecal matter) than the samples at day 0.

The circadian and weather fluctuations in treatment E were reflected by the wide ranges of RH and T values (Figure 1) and affected the water content of samples overtime. In fact, although loss of water (compared to day 0) was observed in samples collected at days 1 and 2, when the weather was dry, samples collected at day 4 showed water gain due to a rain event (whereas a loss of water was observed in samples collected at day 7 due to the drier conditions since the previous sampling). A similar relationship was found in another study [10], which highlighted that the time taken by samples to dry in the field was dependent on weather conditions. Other researchers [19] provided cover during rain episodes to avoid its effect on samples, or artificially poured water on samples to illustrate rain events. Thus, weather conditions, and in particular rain, have an impact on water loss (or gain) and thereby impact the structure of faecal pellets, which needs to be considered when FCM analysis is being conducted.

Although the original FCM values appeared to indicate that FCMs increased over time, there was an overall decrease in adjusted FCM values in comparison to the original ones. The adjustments of FCM values by the mathematical addition of water removed the bias associated with the impact of varying weather conditions. In fact, the mixed effects model analysis detected significant differences in original values over time with all EIAs in most treatments (except in E treatment with 50c and in H with cortisol EIA). However, after the addition of water, no significant variations over time were detected for both the 37e and 50c EIAs. Adjusted cortisol EIA values showed significant variations in treatment E, but not in L or H, which indicates that cortisol EIA might be more affected by changes in metabolites overtime and, hence, are less stable than 37e and 50c.

However, some differences in concentrations may be attributed to intrasample variations in FCM levels (50c > 37e > cortisol EIA), as was previously found in pellets of one defecation [6].

The effect of water (which supports bacterial enzyme activity) on samples is particularly relevant during storage [20,21]. Therefore, we froze the samples for storage to avoid further changes of FCM levels. Whereas some studies reported an increase in FCM values after simulated or natural wet weather [12,22], others could not identify changes after water was poured onto the samples [19]. It was interesting to notice that, in our study, despite the physical change in the pellets due to rain in treatment E, there was no significant variation in the adjusted FCM values measured by the 50c EIA. A study on elephant dung [19] detected that FCM values were stable for eight hours, but increased thereafter in sunny conditions, but less in shade. However, because the samples were not weighed before and after collection, it was not clear from this study whether the differences in the FCM concentrations were artefactual or actual. Another investigation [10] on elephants, which used freeze-dried faeces, found that FCMs were relatively stable for up to 20 h, but decreased thereafter. Studies on the FCMs of jaguars, conducted during a wet and dry season [23], detected no variation over time with both a cortisol EIA and a corticosterone radioimmunoassay (RIA) during the dry season. However, during the wet season, stability was less than one day, and variations were detected thereafter with the cortisol EIA, but not with the corticosterone RIA, which again would confirm our findings that cortisol EIA is more affected by changes in metabolites.

Aside from the effect of rain on FCMs, one important observation is that samples collected at day 4 of E treatment (after the rain event) looked freshly defecated and were heavier than at day 0, indicating absorption of water, as was also observed after jaguar faeces were exposed to rainfall [23]. This observation, combined with the findings on the physical decay of koala pellets during rain events [24], suggests that field collections of pellets should be avoided after rainfall.

The high biological sensitivity of the 50c EIA in measuring koala FCMs was shown in Santamaria et al. [6,9], where the same three EIAs used in the current study were also compared to each other. Here, 50c was shown to be more stable than any of the other EIAs in detecting FCM values before and after the adjustments. Moreover, this study suggests that even if the samples had been freeze-dried, 50c would have still been the most suited, and cortisol the least suited EIA (as also described by Mesa-Cruz et al. [23], who used freeze-dried, homogenised, and pulverised samples). Previous studies [8,25] also highlighted the importance of using species-targeted EIAs after their biological validation, even in cases of related species. In fact, we have shown that, despite all three EIAs measuring FCMs, the highest accuracy that is needed for the detection of stress is only achieved by using the 50c EIA.

More than 1750 papers have been published on FCM studies [26], and the list will increase, with non-invasive research using faecal matter being an ethically responsible, scientifically acceptable, and efficient method [25,27]. However, FCM studies need to be species and location centric because the effect of bacteria on metabolites is not homogeneous across species. Furthermore, stability experiments are only relevant for the species under investigation using a specific EIA because FCM values of the same samples may vary considerably when detected by different EIAs [2,23,28]. For this reason, and given our findings (the effects of environment/rain on pellet structure), we recommend that, in koala studies, only freshly defecated faeces are collected for FCM analyses, despite the increased collection effort for obtaining such samples.

## 5. Conclusions

Here, we have reported on a study that should have detected FCM decay in koalas’ faeces over time. However, to our surprise, findings indicated that changes in FCM levels were mainly driven by water content rather than changes in the structure of the measured metabolites. We highlighted that it is necessary to consider and correct for water loss/gain to determine whether FCM changes are factual or artefactual. Moreover, future studies should attempt to detect changes in gut microbiome in faecal matter and establish whether a correlation between these changes and FCM values exists. However, considering all the variables that can influence the detection of FCMs, our future approach is to restrict stress studies to the analysis of fresh pellets.

Moreover, we have confirmed that the 50c EIA is best suited to determining FCM values in koalas’ faeces. In fact, besides its higher biological sensitivity, FCM levels remained more stable over time in the environmental trial (before and after correction for water loss), when compared with the cortisol and 37e EIAs.

Overall, this and previous research in koala FCMs are important milestones in the development of a methodology to measure stress in koalas using faecal pellets. This methodology will aid managers, policy makers, and those concerned with koala health to understand whether stress can have an impact on koala welfare.

## Data Availability

Raw data are available from corresponding authors upon reasonable request.

## Supplementary Materials

The following are available online at https://www.mdpi.com/article/10.3390/ani11123376/s1, Figure S1: Relationship between water loss and the range of T (temperature in °C) and RH (relative humidity in %) values between two successive samplings in the treatment E (environment). The line indicates a linear prediction with a 95% confidence interval. There was a moderate correlation between mean water loss and ranges of T (Spearman Rank = 0.62, *p* = 0.025) and RH (Spearman Rank = 0.55, *p* = 0.053) values.; Figure S2: Relationship between water loss and the range of RH (relative humidity in %) values between two successive samplings in the treatment H (high temperature, T and high relative humidity, RH). The line indicates a linear prediction with a 95% confidence interval. There was no correlation between water loss and the range of humidity values for H (Spearman Rank=0.068, *p* = 0.692). Table S1: Descriptive statistics of original and adjusted FCM (faecal cortisol metabolite) values (ng/g) for the 50c EIA (enzyme immunoassay) in the three treatments E (environment), L (standard ambient T, 25 °C), and H (high temperature, T and high relative humidity, RH).
