# Supplementary material for: Over Time Decay of Cortisol Metabolites in Faecal Pellets of Koalas in Central Queensland
Source: Animals (Basel). 2021 Nov 25;11(12):3376. doi: 10.3390/ani11123376 (PMC8698095; doi:10.3390/ani11123376)
Supplement: Supplementary file 1 [file animals-11-03376-s001.zip › animals-1455619-supplementary.pdf]

Supplementary material

Over time Decay of Cortisol Metabolites in Faecal Pellets of Koalas in Central Queensland

Flavia Santamaria [1,*], Rolf Schlagloth [1,] Rupert Palme [2] and Joerg Henning [3]

[1] Koala Research-Central Queensland and Flora, Fauna and Freshwater Research, School of Health, Medical and Applied Sciences, Central Queensland University, North Rockhampton, Queensland 4702, Australia; r.schlagloth@cqu.edu.au

[2] Department of Biomedical Sciences, University of Veterinary Medicine, 1210 Vienna, Austria; Rupert.Palme@vetmeduni.ac.at

[3] School of Veterinary Science, The University of Queensland, Gatton, Queensland 4343, Australia; j.henning@uq.edu.au

* Correspondence: f.santamaria@cqu.edu.au

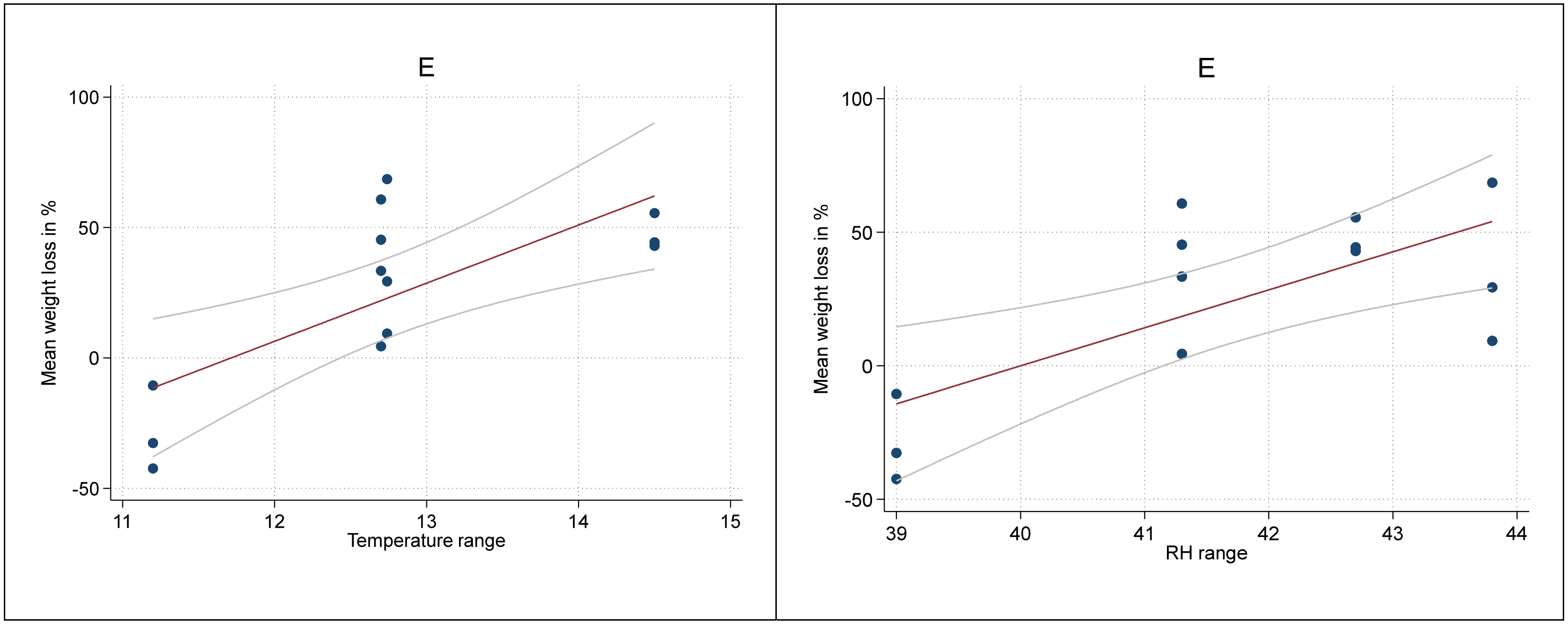

Figure S1. Relationship between water loss and the range of T (temperature in °C) and RH (relative humidity in %) values between two successive samplings in the treatment E (environment). The line indicates a linear prediction with a 95% confidence interval. There was a moderate correlation between mean water loss and ranges of T (Spearman Rank = 0.62, $p = 0.025$) and RH (Spearman Rank = 0.55, $p = 0.053$) values.

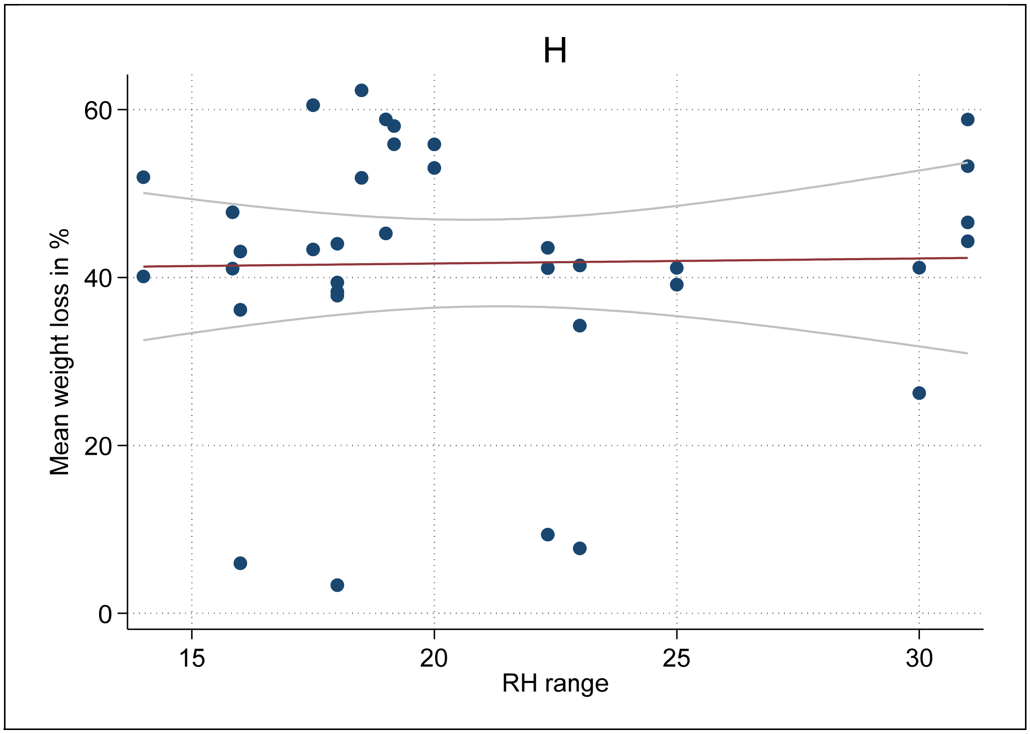

Figure S2. Relationship between water loss and the range of RH (relative humidity in %) values between two successive samplings in the treatment H (high temperature, T and high relative humidity, RH). The line indicates a linear prediction with a 95% confidence interval. There was no correlation between water loss and the range of humidity values for H (Spearman Rank=0.068, p=0.692).

Table S1. Descriptive statistics of original and adjusted FCM (faecal cortisol metabolite) values (ng/g) for the 50c EIA (enzyme immunoassay) in the three treatments E (environment), L (standard ambient T, 25 °C), and H (high temperature, T and high relative humidity, RH).

**Treatments**	**Mean FCMs (ng/g)**		**Median FCMs (ng/g)**		**Min FCMs (ng/g)**		**Max FCMs (ng/g)**	
	**original**	**adjusted**	**original**	**adjusted**	**original**	**adjusted**	**original**	**adjusted**
E	32.35	25.19	26.14	24.25	9.59	6.29	68.05	74.95
L	32.81	21.84	27.47	21.83	11.97	10.67	77.73	38.59
H	30.60	19.66	25.95	17.02	8.99	5.69	66.44	41.87
